# Complete mitochondrial genome of *Geoemyda spengleri*

**DOI:** 10.1080/23802359.2020.1805372

**Published:** 2020-08-07

**Authors:** Bo Zhao, Handong Wang, Jinghong He, Peter Mrope, Yi Mu

**Affiliations:** College of Fisheries, Zhejiang Ocean University, Zhoushan, Zhejiang Province, China

**Keywords:** Mitochondrial genome, *Geoemyda spengleri*, phylogenetic tree

## Abstract

In this study, we determined the complete mitochondrial genome of *Geoemyda spengleri*. The genome was 17,448bp in length and contained 13 protein-coding genes, 22 transfer RNA genes, 2 ribosomal RNA genes, and 1 main non-coding regions. The overall base composition of *G. spengleri* is A 33.67%, T 27.64%, C 25.56%, and G 13.14%, with a highly A + T bias of 61.31%. Here, we describe a phylogenetic analysis of 16 species of Tesudines based on the complete mitochondrial genome, the result showed that *G. japonica* is most closely related to *G. spengleri*. This mitogenome sequence data would play an important role in the investigation of phylogenetic relationship, taxonomic resolution and phylogeography of the Tesudines.

*Geoemyda spengleri*, which belongs to Geoemyda, Batagurinae, Cryotodira, Reptilia. *Geoemyda spengleri* is a second-class protected wildlife in China, wildly distributed in Hunan, Guangdong, Guangxi, etc (Dong 2016). In order to provide useful information for the future research of genetic diversity and phylogenetic,we determined the complete mitochondrial genome of *G. spengleri* (GenBank accession number KU641028).

*Geoemyda spengleri* were obtained from the mountain forest. We took the muscle tissue from the severed tail and disinfected the turtle’s wound with alcohol. Initially, the turtle were identified based on both the morphologic features and the COX1 mitochondrial gene. Tissue samples for molecular analysis were reserved in 95% ethanol.Whole genomic DNA was extracted from muscle tissue of individual specimens using the phenol-chloroform method. Polymerase chain reaction (PCR) was performed. The PCR products were sequenced by Sangon Biotech, Shanghai, China.

Sequences were assembled using Generious 4.5.3 (http://www.geneious.com). BioEdit 7.0 (Hall, 1999) was used for sequence alignment. The neighbor-joining (NJ) (Saitou and Nei 1987) and maximum likelihood (ML) (Tamura et al. 2004) methods were used to construct the phylogenetic trees. The NJ trees were obtained with 10,000 bootstrap replications using MEGA7.0 (Kumar et al. 2016).

The complete mitochondrial genome of *G. spengleri* is 17,448bp in length and consists of 13 protein-coding genes, 22 transfer RNA genes (tRNA), 2 ribosomal RNA genes (rRNA) and 1 control regions (CR) (Table 1). The mitogenome base composition was A33.67%, T 27.64%, C25.56%, and G 13.14%, A + T content (61.31%) was much higher than the G + C content (38.69%), in common with other vertebrate mitogenomes. Except for eight tRNA (tRNA^Ser^, tRNA^Pro^, tRNA^Glu^, tRNA^Tyr^, tRNA^Cys^, tRNA^Asn^, tRNA^Ala^, tRNA^Gln^) and the ND6 genes encoded on the L-strand, the other genes are encoded on the H-strand.Ten protein-coding genes(ND1, COX2, ATP8, ATP6, COX3, ND3, ND4L, ND4, ND5, CYTB) start with an ATG initiation codon, while ND2 uses ATA as the initiation codon, COX1 uses GTG as the initiation codon, and ND6 uses CCT as the initiation codon. Six protein-coding genes (COX2, ATP8, ATP6, ND4L, ND4 and ND5) use TAA as the termination codon; three protein-coding genes (ND1, ND2 and ND3) use TAG as the termination codon, while COX3 and ND6 share the termination codon CAT; COX1 use AGG as the termination codon, CYTB uses the incomplete stop codon T. The two ribosomal RNA genes, 12SrRNA (964 bp) is located between tRNA^Phe^ and tRNA^Val^ genes, and 16SrRNA (1681 bp) is located between tRNA^Val^ and tRNA^Leu^ genes. The complete mitochondrial genome of *G. spengleri* has the only control region (1937 bp) gene located between tRNA^Pro^ and tRNA^Phe^.

**Table 1.** Mitochondrial genome characteristics of the *Geoemyda spengleri*.

Gene	Position		Size(bp)		Codon		Intergenic nucleotides	Strand
	From	to	Nucleotide	Amino acid	Initiation	Stop		
tRNA^Phe^	1	70	70					H
12S rRNA	71	1034	964					H
tRNA^Val^	1035	1104	70					H
16S rRNA	1105	2715	1681					H
tRNA^Leu^	2716	2790	75					H
ND1	2791	3762	972		ATG	TAG		H
tRNA^Ile^	3762	3831	70				–1	H
tRNA^Gln^	3831	3901	71				–1	L
tRNA^Met^	3902	3971	70					H
ND2	3972	5012	1041		ATA	TAG		H
tRNA^Trp^	5011	5086	76				–2	H
tRNA^Ala^	5088	5156	69				1	L
tRNA^Asn^	5158	5230	73				1	L
tRNA^Cys^	5257	5322	66				26	L
tRNA^Tyr^	5323	5393	71					L
COI	5395	6942	1548		GTG	AGG	1	H
tRNA^Ser^	6943	7004	62					L
tRNA^Asp^	7007	7076	70				2	H
COII	7077	7763	687		ATG	TAA		H
tRNA^Lys^	7765	7837	73				1	H
ATP8	7839	8006	168		ATG	TAA	1	H
ATP6	7985	8680	696		ATG	TAA	–21	H
COIII	8680	9463	784		ATG	CAT		H
tRNA^Gly^	9464	9531	68					H
ND3	9532	9883	352		ATG	TAG		H
tRNA^Arg^	9881	9951	70				–3	H
ND4L	9952	10,248	297		ATG	TAA		H
ND4	10,242	11,618	1377		ATG	TAA	–7	H
tRNA^His^	11,635	11,703	69				6	H
tRNA^Ser^	11,704	11,767	64					H
tRNA^Leu^	11,769	11,840	72				1	H
ND5	11,841	13,634	1794		ATG	TAA		H
ND6	13,630	14,154	525		CCT	CAT	–5	L
tRNA^Glu^	14,155	14,222	68					L
CYTB	14,227	15,370	1144		ATG	T	4	H
tRNA^Thr^	15,371	15,441	71					H
tRNA^Pro^	15,443	15,511	69				72	L
Control Region	15,512	17,448	1937					H

In this study, *Geoemyda japonica* is most closely related to *G. spengleri*, *Chelydra serpentina* was placed at the most basal position forming an individual clade, while other species formed another large cluster. *Cuora pani* was grouped with *Cuora trifasciata,Cuora flavomarginata, Pyxidea mouhotii, Cuora galbinifrons* and *Cuora amboinensis*, *Sacalia bealei, Heosemys depressa* and *Cyclemys dentata* were clustered together,while the other three turtles were formed a clade (Figure 1). *Geoemyda spengleri* is one of the endemic species in china. We expect that the present result will facilitate the further investigations of phylogenetic relationship,taxonomic resolution and phylogeography of the Tesudines.

**Figure 1.**
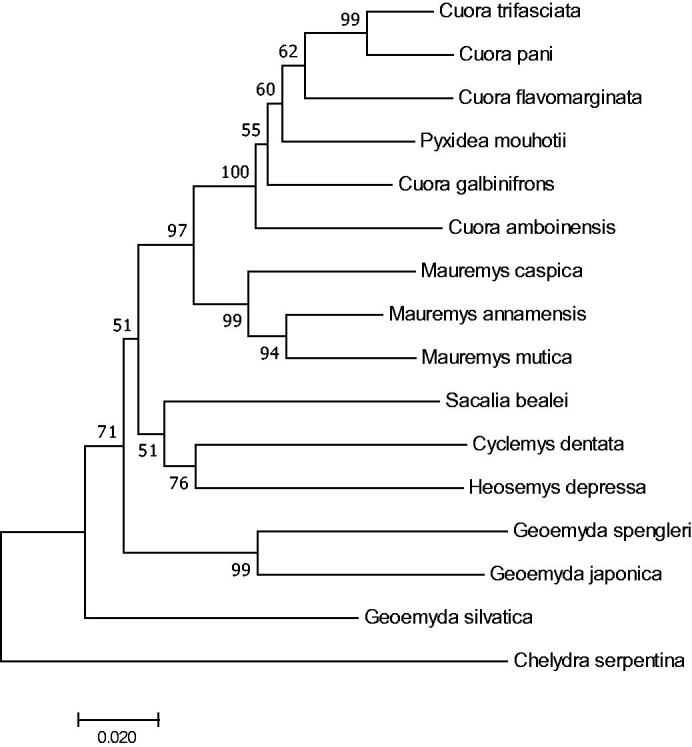
Molecular phylogenetic tree based on mitochondrial partial cytb gene for cytochrome b.
